# Safety profile of TNF- alpha Inhibitors in pediatric patients: A post-marketing surveillance study based on the FAERS database

**DOI:** 10.1371/journal.pone.0328465

**Published:** 2025-12-02

**Authors:** Yanmo Yang, Peng Wang, Qin-xuan Li

**Affiliations:** Department of pharmacy, Changde Hospital, Xiangya School of Medicine, Central South University (The first people’s hospital of Changde city), Changde, Hunan, China; Istituto Dermopatico dell'Immacolata (IDI)-IRCCS, ITALY

## Abstract

This study systematically evaluated the safety profile of tumor necrosis factor-alpha (TNF-α) inhibitors in pediatric patients using data from the U.S. Food and Drug Administration (FDA) Adverse Event Reporting System (FAERS) from Q1 2004 to Q3 2024.Through disproportionality analysis of adverse event (AE) reports for infliximab, etanercept, adalimumab, golimumab and certolizumab, we identified 852 significant safety signals spanning 27 system organ classes (SOCs). The most frequently reported SOCs included General Disorders and Administration Site Conditions (12,940 cases), Injury, Poisoning, and Procedural Complications (5,503 cases), and Gastrointestinal Disorders (4,346 cases).Key findings revealed that infection-related AEs and injection-site reactions were the predominant safety concerns. The median onset time of AEs was 235 days (IQR: 46–832 days), with 19.8% of cases occurring within the first month of treatment. Notably, 25.5% of reported AEs required hospitalization, while fatal and life-threatening outcomes accounted for 0.9% and 1.4% of cases, respectively. This comprehensive analysis confirms the multisystemic involvement and prolonged latency of TNF-α inhibitors-associated AEs in pediatric populations. While these agents remain vital for managing chronic inflammatory diseases, the findings advocate for enhanced clinical vigilance. We propose a tiered monitoring protocol prioritizing infection surveillance (e.g., serial inflammatory markers), systematic injection-site evaluations, and longitudinal organ function assessments, particularly during the initial treatment phase, to optimize therapeutic risk-benefit ratios.

## Introduction

As significant biologic therapeutics, tumor necrosis factor-alpha (TNF-α) inhibitors have achieved groundbreaking progress in treating pediatric chronic inflammatory diseases, including inflammatory bowel disease (IBD) and juvenile idiopathic arthritis (JIA) [1]. According to the European Medicines Agency (EMA) 2022 Annual Report, 11.9% annual growth rate in pediatric TNF-α inhibitors usage from 2015 to 2022 [2]. Notably, adalimumab and infliximab dominate pediatric TNF-α inhibitors prescriptions, collectively accounting for 73.8% of total usage [3]. Despite their therapeutic efficacy, TNF-α inhibitors are associated with profound immunosuppression, which escalates susceptibility to infections [4]. Furthermore, the unique physiological characteristics and developmental stage-specific vulnerabilities of children necessitate a thorough understanding of their safety profiles.

Current pediatric pharmacovigilance systems grapple with two critical limitations: (1) Inadequate clinical trial representation: Children constitute less than 4% of participants in pre-marketing drug trials [5], leading to a paucity of robust safety data. (2) Systematic underdetection: Spontaneous reporting systems exhibit persistent underreporting of pediatric AEs [6]. Emerging evidence reveals an inverse correlation between age and injection-site reaction incidence, alongside age-dependent variations in biologic sensitivity [7]. These factors collectively obscure a comprehensive understanding of AE patterns in pediatric populations. Traditional safety assessments often extrapolate data from adult populations, a practice increasingly recognized as insufficient given the documented age-related differences in AE incidence and manifestation. This gap highlight the urgent need for dedicated pediatric pharmacovigilance studies that account for these developmental variations. While existing pediatric safety data on TNF-α inhibitors are available, they frequently lack the granularity and comprehensive, real-world scope necessary to identify subtle yet significant AE signals across various age level.

FAERS is a globally recognized pharmacovigilance database, provides comprehensive post-marketing safety surveillance data, particularly identifying emerging safety concerns in pediatric populations. Its vast repository of real-world post-marketing data allows for the detection of rare adverse events and the identification of trends that might not be apparent in controlled clinical trials. Utilizing a comparative, age-stratified approach with the FAERS database, this study aims to comprehensively characterize the spectrum, frequency, and severity of adverse events associated with TNF-α inhibitors (infliximab, etanercept, adalimumab, golimumab, and certolizumab) in pediatric patients from Q1 2004 to Q3 2024. Our objective is also to identify key demographic and clinical factors influencing these events, thereby providing valuable insights for age-specific risk mitigation strategies and monitoring protocols.

## Materials and methods

### Data sources

This study analyzed safety data for five TNF-α inhibitors: infliximab (approved 1998), etanercept (approved 1998), adalimumab (approved 2002), golimumab (approved 2009), and certolizumab (approved 2008). Data were extraction from FAERS from Q1 2004 to Q3 2024. Reports prior to Q1 2004 were excluded to minimize bias from the extended market exposure of earlier-approved drugs (e.g., infliximab, etanercept, adalimumab). For golimumab and certolizumab, only post-approval reports were included (i.e., no pre-2009 or pre-2008 data, respectively). Medication identification utilized standardized Medical Subject Headings (MeSH) terms including: Infliximab (Remicade), Etanercept (Enbrel, Erelzi), Adalimumab (Humira, mjevita, Cyltezo),Golimumab (Simponi),Certolizumab (Cimzia). Quarterly FAERS datasets comprised seven structured tables:DEMO(Demographic information),REAC(Adverse event documentation),DRUG(Medication usage records),OUTC(Treatment outcomes),RPSR(Report sources),THER(Treatment initiation/cessation dates),INDI(Indication details).In this study, drug reports were restricted medications classified as the “Primary Suspect (PS)” in causality assessment. Pediatric data were extracted from the DEMO table for cases with age < 18 years, excluding records with missing or ambiguous age data. Age was extracted in years, and when reported in months, weeks, or days, we converted to years for consistency (e.g., 6 months = 0.5 years). Records with missing age data, ambiguous units, or implausible values (e.g., negative or non-numeric entries) were excluded from age-stratified analyses. For age-stratified analyses, cases with incomplete age information were further excluded.

### Data extraction and processing

AEs in the FAERS database were coded using MedDRA version 26.1 PT codes and were systematically classified in the REAC table into broad SOC and specific PT categories. As FAERS is a voluntary reporting system for healthcare professionals and consumers, it contains numerous duplicate reports and cases with missing information. To identify and remove duplicate reports, this study selected the PRIMARYID, CASEID, and FDA_DT fields from the DEMO table and sorted them. When CASEID values were identical, only the report with the most recent FDA_DT date was retained. When both CASEID and FDA_DT values were identical, the report with the highest PRIMARYID value was preserved. Additionally, since the first quarter of 2004, each quarterly data package has included a list of deleted reports. After deduplication, reports corresponding to CASEIDs listed in the deleted reports list were excluded.

To mitigate indication bias (misclassification of therapeutic indications as ADR), PTs corresponding to FDA-approved indications for TNF-α inhibitors (e.g.,IBD for infliximab) were excluded from the analysis. This multi-step approach enhanced data validity while preserving critical pharmacovigilance signals.

**Fig 1:**
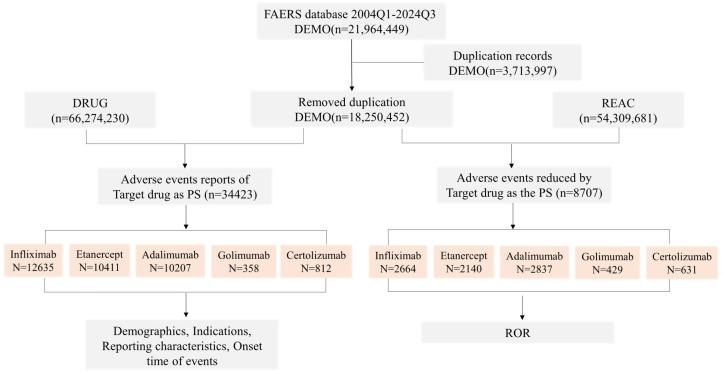
Flow Chat of Data Extraction and Selection from the FAERS Database. This flow chat showing the process of data extraction and selection from the FAERS database for the period from Q1 2004 to Q3 2024. ROR, reporting odds ratio; PS, primary suspected drugs.

Off-label use filtering. Reports in which “Off-label use” was the only PT were retained for two reasons: (1) golimumab lacks paediatric approval, so any paediatric exposure is inherently off-label; removing these reports would eliminate the only real-world exposure data for this drug in children. (2) To keep cross-drug comparability, the same rule was applied to infliximab, etanercept, adalimumab and certolizumab. Reports coded solely to the ‘Product Issues’ SOC were excluded from disproportionality analysis because they reflect hardware or administration errors, not medicinal adverse events. The process of data extraction, deduplication, and selection of pediatric cases is summarized in Fig 1.

### Data analysis and statistics

This study employed a two-phase analytical approach to evaluate the safety profiles of TNF-α inhibitors in pediatric populations. First, a comprehensive descriptive analysis was conducted to characterize ADE reports for five TNF-α inhibitors, encompassing report frequencies, demographic distributions (gender, age subgroups), clinical outcomes, and reporter classifications. Second, disproportionality analysis was performed to identify ADR risk signals, using the ROR and its 95% CI as statistical metrics. The ROR was employed as the primary disproportionality metric because it is widely used, computationally efficient, and well understood within pharmacovigilance. While it is susceptible to notoriety bias and lacks control for drug exposure or duration. To reduce false positives, we only retained signals with ≥3 reports and a lower 95% CI > 1. The fourfold contingency table for disproportionality analysis was constructed as follows [8]:


$$\text{ROR}=\;\frac{\text{ad}}{\text{bc}}$$

$$\text{95}\%\text{CI}=e^{InROR\pm \text{1}.\text{9}\text{6}\sqrt{\frac{\text{1}}{a}+\frac{\text{1}}{b}+\frac{\text{1}}{c}+\frac{\text{1}}{d}}}$$

where:

a = number of target ADE reports for the target drug

b = number of non-target ADE reports for the target drug

c = number of target ADE reports for comparator drugs

d = number of non-target ADE reports for comparator drugs

Higher ROR values indicate stronger signal strength, reflecting greater likelihood of statistical association between the target drug and specific AE. The risk-signal detection ratio (RSR) was defined as the ratio of a risk signal to all PT reports in each drug.. ADR signals were prioritized based on two criteria: (1) highest pediatric ADE report volumes and (2) most frequent ADR signal recurrence across all five TNF-αinhibitors. This dual-criterion approach enabled identification of both high-prevalence and pan-class safety concerns.

### Subgroup analysis

In subgroup analyses, risk signal outcomes were evaluated at the SOC level across age-stratified cohorts. Patients were categorized into three age groups: 0–3 years, 4–11 years, and 12–17 years. Results for each age group were ranked by case frequency, and the top 10 AEs associated with TNF-α inhibitors were systematically compared across these age strata.

### Sensitivity analysis

To further assess potential time bias, we stratified the analysis into two periods: Q1 2004–Q4 2013 (early phase) and Q1 2014–Q3 2024 (recent phase). The 50 most frequently reported preferred terms were treated as paired observations and compared across the two eras using the Wilcoxon signed-rank test (two-tailed).

## Results

### Data baseline and demographic characteristics

A total of 1,403,245 adverse drug event (ADE) reports associated with five TNF-α inhibitors were extracted from the FAERS between Q1 2004 and Q3 2024, following deduplication procedures. These reports designated one of the following agents as the Primary Suspect Drug (PS),including infliximab (169,243 total reports; 12,635 pediatric cases, 7.5% of total), etanercept (505,508 total reports; 10,411 pediatric cases, 2.1% of total), adalimumab (604,951 total reports; 10,207 pediatric cases, 1.7% of total), golimumab (45,643 total reports; 358 pediatric cases, 0.8% of total), and certolizumab pegol (77,900 total reports; 812 pediatric cases, 1.0% of total). Among the 34,423 pediatric ADE reports analyzed, gender distribution varied by agent. A marginal male predominance was observed among infliximab users (49.4% male vs. 49.1% female), female predominance characterized other agents (etanercept: 67.5% female; adalimumab: 53.3%; golimumab: 62.3%; certolizumab: 48.0%). Significant age-dependent variations emerged across three pediatric subgroups: infants/toddlers (0–3 years), children (4–11 years), and adolescents (12–17 years), with distinct AE reporting patterns observed for each TNF-α inhibitor.Healthcare professionals constituted the primary source of reports(63.3%), followed by consumers submissions (35.0%).Severe outcomes—defined as events involving death, life-threatening conditions, disability, or hospitalization—exhibited agent-specific disparities.Infliximab demonstrated the highest proportion of severe outcomes (24.6% of pediatric cases), followed by golimumab (18.9%), certolizumab pegol (15.2%), adalimumab (12.8%), and etanercept (9.1%). Comprehensive demographic characteristics and clinical outcome distributions are detailed in Table 1.

**Table 1:** Characteristics of pediatric adverse event reports for TNF-α inhibitors.

Characteristics	TNF inhibitors (N = 34,423)	Infliximab (n = 12,635)	Etanercept (n = 10,411)	Adalimumab (n = 10,207)	Golimumab (n = 358)	Certolizumab (n = 812)
**gender, n (%)**						
**Male**	14409(41.9)	6239(49.4)	3133(30.1)	4558(44.7)	126(35.2)	353(43.5)
**Female**	19287(56.0)	6209(49.1)	7028(67.5)	5437(53.3)	223(62.3)	390(48.0)
**Unknown**	727(2.1)	187(1.5)	250(2.4)	212(2.1)	9(2.5)	69(8.5)
**age(year),n (%)**						
**0-3**	2453(7.1)	672(5.3)	739(7.1)	363(3.6)	151(42.2)	528(65.0)
**4-11**	8296(24.1)	2532(20.0)	3434(33.0)	2239(21.9)	57(15.9)	34(4.2)
**12-17**	23674(68.8)	9431(74.7)	6268(59.9)	7605(74.5)	150(41.9)	250(30.8)
**Type of reporter, n (%)**						
**Physician**	12244(35.6)	5011(39.7)	5109(49.1)	1673 (16.4)	161(45.0)	290 (35.7)
**Pharmacist**	1078(3.1)	363(2.9)	388(3.7)	261 (2.6)	35(9.8)	31 (3.8)
**Other health professional**	8462(24.6)	5151(40.8)	1840(17.7)	1180(11.6)	80(22.4)	211(26.0)
**consumer**	12060(35.0)	2050(16.2)	2875(27.6)	6792 (66.5)	78(21.8)	265 (32.6)
**Unknown or other**	579(1.7)	60(0.5)	199(1.9)	301(2.9)	4(1.1)	15(1.8)
**Outcomes, n (%)**						
**Death**	300(0.9)	149(1.2)	68(0.7)	74(0.7)	4(1.1)	5 (0.6)
**Life-Threatening**	490(1.4)	294(2.3)	82(0.8)	99(1.0)	8(2.2)	7(0.9)
**Disability**	173(0.5)	57(0.4)	46(0.4)	68(0.7)	2(0.6)	–
**Hospitalization**	8771(25.5)	5432(43.0)	712(6.8)	2319(22.7)	111(31.0)	197 (24.3)
other*	9889(28.7)	5938(47.0)	1576(15.1)	1798(17.6)	136(38.0)	441(54.3)
**Unknown**	14800(43.0)	765(6.1)	7927(76.1)	5849(57.3)	97(27.1)	162 (20.0)

Footnotes: Other events: Includes congenital anomalies, permanent disabilities, and other medically significant events not categorized elsewhere.

### Characteristics of disproportionality analysis for TNF-α inhibitors

**Fig 2:**
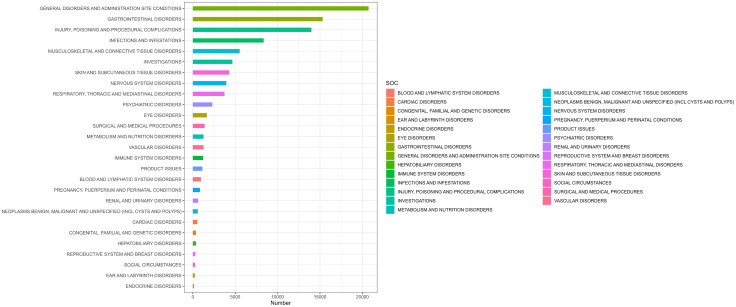
Distribution of AE Signals by SOC. This bar chart showing the distribution of AE signals categorized by their respective SOCs based on reports from the FAERS database. The x-axis represents the number of reported adverse events, while the y-axis lists the various SOC categories.

A total of 852 AE signals were detected for the five TNF-α inhibitors in pediatric populations, distributed across 27 SOCs (Fig 2; S1 Table). By report frequency, the top three SOCs in pediatric populations were infections and infestations (169 cases, 19.84%), gastrointestinal disorders (106 cases, 12.44%), and general disorders and administration site conditions (81 cases, 9.51%). In contrast, the leading SOCs across all age groups were infections and infestations (492 cases, 16.71%), neoplasms benign, malignant, and unspecified (incl. cysts and polyps) (364 cases, 12.36%), and surgical and medical procedures (335 cases, 11.37%). These findings highlight age-related disparities in AE profiles, with pediatric populations exhibiting heightened vulnerability to infection-related and gastrointestinal complications compared to broader age demographics.

**Fig 3:**
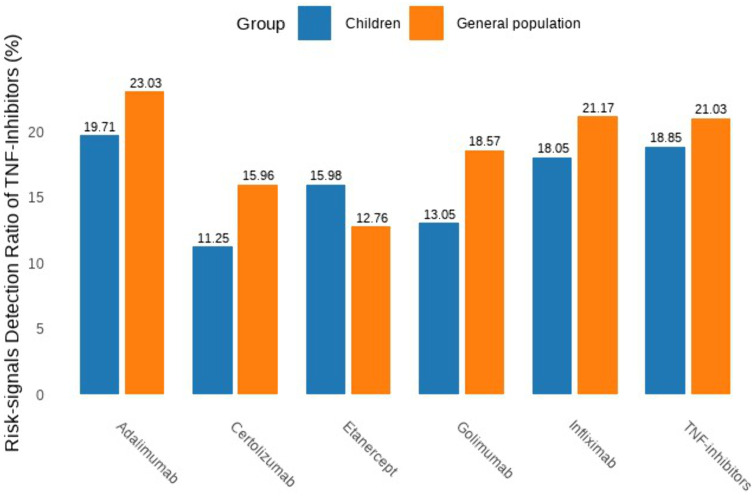
Risk-signal detection ratio for each TNF-α inhibitors. The result is the ratio of the number of risk signals involved in each kind of TNF-α inhibitors to the total recorded signals.

Among 14,002 PTs identified in all age groups, 4,520 (32.3%)were detected in pediatric populations. Using predefined detection thresholds(reporting odds ratio(ROR) >2, 95% confidence interval (CI)>1, ≥ 3 cases), 2,944 potential risk signals were detected overall, with 852 (28.9%) identified in children. Comparative analysis demonstrated a significantly lower risk signal detection rate (RSR) in children (18.85% [852/4,520]) compared to the all-age groups (21.03% [2,944/14,004]) (Fig 3). In pediatric analyses, adalimumab showed the highest proportion of risk-associated PTs (19.71%), followed by infliximab (18.05%). Notably, etanercept exhibited a higher risk PT proportion in children compared to all-age data (15.98% vs. 12.76%), while the remaining four TNF-α inhibitors demonstrated significantly lower pediatric risk PT proportions than population-wide results.These findings highlight age- and agent-dependent variations in TNF-α inhibitor safety profiles.

### Risk signal distribution in pediatric

**Fig 4:**
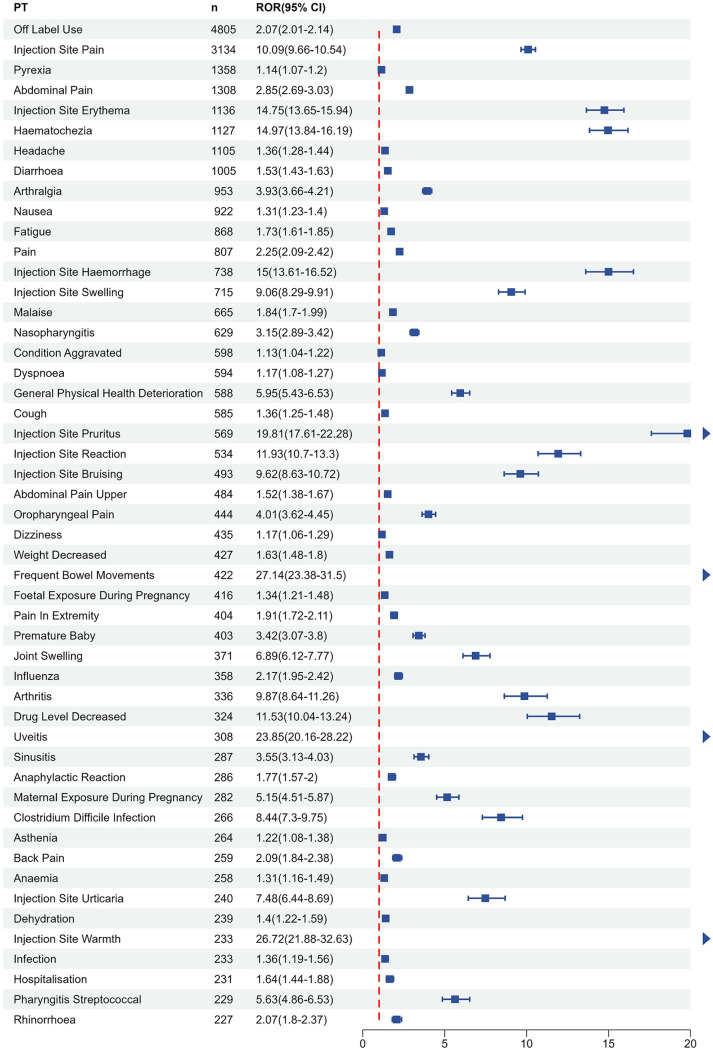
The top 50 adverse events reported by frequency of TNF-α inhibitors in children. The x-axis represents the ROR, indicating the strength of the association between each AE and TNF-α inhibitor treatment. The number of reports (n) listed alongside each PT.

A total of 852 potential risk signals were identified in pediatric patients exposed to five TNF-α inhibitors, with the top 50 signals in Fig 4 franked by report frequency after excluding signals unrelated to adverse drug reactions (e.g., those associated with therapeutic indications). The most frequently reported PTs were off-label use (4,805 cases), injection site pain (3,134 cases), and pyrexia (1,358 cases). The three PTs with the highest RORs were increased bowel frequency (ROR = 27.14, 95% CI 19.85–37.12), injection site warmth (ROR = 26.72, 95% CI 20.34–35.09), and uveitis (ROR = 23.85, 95% CI 17.63–32.26). At the SOC distribution, the highest report volumes occurred in general disorders and administration site conditions (12,940 cases), injury, poisoning, and procedural complications (5,503 cases), and gastrointestinal disorders (4,346 cases), collectively representing 48.7% of all SOC-classified reports, indicating a predominant systemic impact of TNF-α inhibitors in pediatric populations.

### Risk signal profile in pediatric

We presented the ROR of the top 50 risk signals in Table 2 for pediatrics, alongside a systematic safety analysis of TNF-α inhibitors based on adverse event reports, including both high-frequency and high-risk signal patterns.

**Table 2:** The ROR of the top 50 AE reports in children.

SOC	PT	Adalimumab		Certolizumab		Etanercept		Golimumab		Infliximab	
		N	ROR(95% CI)	N	ROR(95% CI)	N	ROR(95% CI)	N	ROR(95% CI)	N	ROR(95% CI)
General disorders and dministration site conditions	injection site pain	1546	12.46(11.78-13.17)	13	1.04 (0.6 - 1.79)	1569	16.41 (15.51 −17.35)	5	1 (0.42 - 2.42)	1	0.01 (0 - 0.04)*
	pyrexia	538	1.36 (1.24 −1.48)	25	0.76(0.51-1.13)	463	1.49 (1.35 −1.63)	6	0.46 (0.21 −1.02)	326	0.73 (0.65 - 0.81)
	injection site erythema	372	9.87(8.84- 11.03)	3	0.84 (0.27 - 2.61)	759	31.69 (29.1 - 34.52)	0	/	2	0.04 (0.01 - 0.16)*
	fatigue	409	2.42(2.19-2.68)	17	1.2 (0.74- 1.93)	246	1.82 (1.61 - 2.07)	8	1.42 (0.71 - 2.85)	188	0.98 (0.84 - 1.13)*
	pain	306	2.48(2.21 - 2.78)	13	1.25(0.73-2.16)	356	3.71 (3.34 - 4.13)	7	1.7 (0.81 - 3.58)	125	0.88 (0.74 - 1.06)*
	injection site haemorrhage	557	29.8(26.9-33.02)	2	0.87 (0.22 - 3.49)*	177	8.9 (7.61 - 10.41)	2	2.2 (0.55 - 8.84)*	0	/
	injection site swelling	188	5.47 (4.71 −6.36)	7	2.32 (1.1 - 4.87)	519	23.4(21.19-25.84)	0	/	1	0.02 (0 - 0.17)*
	malaise	243	1.97(1.73-2.24)	5	0.49(0.2-1.17)*	263	2.73(2.42-3.09)	4	0.98 (0.37-2.62)*	150	1.08 (0.92 - 1.27)
	condition aggravated	154	0.86 (0.74 - 1.01)*	9	0.62(0.32-1.19)	285	2.08 (1.85 - 2.34)	0	/	150	0.76 (0.65 - 0.89)
	general physical health deterioration	33	0.8(0.57- 1.12)*	0	/	32	0.99 (0.7 - 1.4)*	0	/	523	14.51 (13.16-15.99)
	injection site pruritus	169	10.54 (8.94 - 12.41)	3	1.97 (0.63 - 6.12)	394	41.58 (36.74 −47.04)	0	/	3	0.14 (0.05 - 0.44)*
	injection site reaction	143	6.79 (5.71 −8.08)	4	2.12 (0.79 −5.66)	383	29.11(25.85 −32.77)	0	/	4	0.15 (0.06 - 0.41)*
	injection site bruising	155	6.98 (5.9 - 8.24)	0	–	337	22.67(20.06-25.62)	1	1.26 (0.18-8.95)*	0	/
	asthenia	136	1.9 (1.6 - 2.26)	5	0.84(0.35-2.02)	51	0.89 (0.67-1.17)*	0	/	72	0.89 (0.71 - 1.13)*
	injection site urticaria	129	10.6(8.78-12.79)	0	/	111	11.34(9.29-13.86)	0	/	0	/
	injection site warmth	44	7.29 (5.33 - 9.98)	1	1.84 (0.26 - 13.07)*	187	66.83 (54.86 −81.42)	0	/	1	0.13 (0.02 - 0.95)*
Injury, poisoning and procedural complications	off label use	534	0.63 (0.58 - 0.69)*	71	1.05 (0.83 - 1.33)	1459	2.34 (2.22 - 2.47)	91	3.61 (2.91 - 4.48)	2650	3.15 (3.02 - 3.28)
	foetal exposure during pregnancy	98	0.93 (0.76 - 1.13)*	165	20.94 (17.85 −24.56)	5	0.06 (0.02 - 0.14)	7	2.06 (0.98 - 4.33)	141	1.22 (1.03 - 1.44)
	maternal exposure during pregnancy	10	0.45 (0.24 - 0.84)*	264	203.67 (176.89 - 234.49)	2	0.11 (0.03 - 0.46)*	0	/	6	0.24 (0.11 - 0.54)*
Gastrointestinal disorders	abdominal pain	430	2.65(2.41 - 2.92)	12	0.87(0.5-1.54)	81	0.61 (0.49 - 0.76)*	2	0.37 (0.09 - 1.47)*	783	4.56 (4.24-4.91)
	haematochezia	261	6.7 (5.89 - 7.61)	5	1.43(0.59-3.43)	17	0.5 (0.31 - 0.8)*	5	3.61 (1.5 - 8.7)	839	26.37(24.27-28.66)
	diarrhoea	396	1.79(1.62-1.98)	15	0.81(0.49-1.35)	174	0.98 (0.84-1.14)*	5	0.68 (0.28 −1.65)	415	1.69 (1.53-1.87)
	abdominal pain upper	225	2.11(1.85 - 2.41)	5	0.56 (0.23 - 1.35)	160	1.89 (1.62 - 2.22)	2	0.57 (0.14 - 2.27)*	92	0.76 (0.62 - 0.93)
	frequent bowel movements	102	9.75(7.91-12.03)	4	4.09 (1.53 - 10.95)	3	0.31 (0.1 - 0.98)*	0	/	313	41.4 (35.7 - 48.01)
Musculoskeletal and connective tissue disorders	arthralgia	297	3.33 (2.96 - 3.75)	9	1.18 (0.62 - 2.28)	419	6.19 (5.6 - 6.84)	7	2.33 (1.11 - 4.91)	221	2.2 (1.92 - 2.52)
	pain in extremity	161	2.23 (1.91 - 2.62)	3	0.5 (0.16 - 1.54)*	189	3.37 (2.91 - 3.9)	1	0.42 (0.06 - 2.97)*	50	0.61 (0.46 - 0.8)*
	joint swelling	105	4.81 (3.94 - 5.87)	1	0.53 (0.07 - 3.73)*	231	15.06 (13.06 −17.35)	1	1.33 (0.19 - 9.44)	33	1.29 (0.91 - 1.82)
	arthritis	103	6.91 (5.63 - 8.47)	0	/	159	14.57 (12.29 −17.27)	5	9.48 (3.93 - 22.87)	69	4.01 (3.14 - 5.12)
	back pain	88	2.06 (1.67 - 2.55)	2	0.56 (0.14 - 2.25)*	112	3.38 (2.8 - 4.09)*	2	1.42 (0.35 - 5.69)	55	1.15 (0.88 - 1.5)
Infections and infestations	nasopharyngitis	209	2.93 (2.55 - 3.37)	9	1.49 (0.78 - 2.87)	292	5.35 (4.75 - 6.03)	7	2.94 (1.4 - 6.18)	112	1.38 (1.14 - 1.66)
	influenza	120	2.11 (1.76 - 2.53)	5	1.05 (0.44 - 2.54)	136	3.06 (2.58 - 3.64)	5	2.67 (1.11 - 6.43)	92	1.44 (1.17 - 1.78)
	sinusitis	115	3.98 (3.29 - 4.81)	3	1.21 (0.39 - 3.75)	126	5.6 (4.67 - 6.72)	0	/	43	1.28 (0.95 - 1.74)
	clostridium difficile infection	56	4.08 (3.11 - 5.35)	2	1.7 (0.42- 6.8)*	1	0.09 (0.01 - 0.62)*	1	2.14 (0.3 - 15.22)*	206	16.72 (14.29 - 19.56)
	infection	89	1.55 (1.26 - 1.92)	7	1.48 (0.71 - 3.12)	72	1.59 (1.26 - 2.01)	5	2.68 (1.11 - 6.45)	60	0.93 (0.72 - 1.21)*
	pharyngitis streptococcal	82	5.27 (4.2 - 6.61)	1	0.73 (0.1 - 5.21)*	102	8.56 (6.97 - 10.51)	0	/	44	2.45 (1.81 - 3.31)
	Viral infection	66	1.35 (1.06 - 1.72)	7	1.74 (0.83 - 3.67)*	83	2.18 (1.75 - 2.71)	4	2.52 (0.94 - 6.72)*	64	1.18 (0.92 - 1.51)*
Respiratory, thoracic and mediastinal disorders	dyspnoea	106	0.62(0.51-0.75)*	3	0.21 (0.07 - 0.67)*	52	0.38 (0.29 - 0.5)*	3	0.54 (0.17 - 1.68)*	430	2.35 (2.13 - 2.59)
	cough	203	1.4(1.22- 1.61)	8	0.67 (0.33 - 1.34)*	226	2 (1.75 - 2.28)	2	0.42 (0.11 - 1.69)*	146	0.9 (0.76 - 1.06)*
	oropharyngeal pain	180	4.5 (3.87 - 5.24)	8	2.31 (1.15 - 4.63)	182	5.82 (5 - 6.77)	3	2.18 (0.7 - 6.79)	71	1.52 (1.2 - 1.93)
	rhinorrhoea	79	2.09 (1.67 - 2.62)	1	0.32 (0.04 - 2.26)*	127	4.4 (3.67 - 5.26)	2	1.61 (0.4 - 6.44)	18	0.42 (0.26 - 0.66)
Nervous system disorders	headache	439	1.61(1.47-1.78)	10	0.44 (0.24 - 0.82)*	410	1.92 (1.74 - 2.12)	5	0.56 (0.23 - 1.34)*	241	0.78 (0.69 - 0.89)
	dizziness	177	1.43(1.24-1.67)	8	0.79 (0.39 - 1.57)*	122	1.25 (1.05 - 1.5)	4	0.99 (0.37 - 2.65)*	124	0.9 (0.75 - 1.07)
Investigations	weight decreased	170	1.93 (1.65 - 2.24)	16	2.19 (1.34 - 3.58)	47	0.66 (0.5 - 0.88)	4	1.38 (0.52 - 3.67)	190	1.95 (1.69 - 2.25)
	drug level decreased	72	5.4 (4.24 - 6.87)	/	/	/	/	2	4.32 (1.08 - 17.32)*	250	22 (18.98 - 25.51)
Pregnancy, puerperium and perinatal conditions	premature baby	39	0.88 (0.64 - 1.21)	193	62.28 (53.5 - 72.5)	55	1.6 (1.22 - 2.09)	4	2.81 (1.05 - 7.5)	112	2.36 (1.95 - 2.85)
Eye disorders	uveitis	145	20.96 (17.34 −25.35)	4	5.32 (1.99 - 14.24)	82	13.07 (10.33 −16.53)	15	52.09 (31.07 −87.33)	62	6.69 (5.14 - 8.72)
Immune system disorders	anaphylactic reaction	15	0.26(0.16-0.44)	5	1.1(0.46 - 2.64)	11	0.25(0.14-0.45)*	/	/	255	4.41 (3.88 - 5.01)
Blood and lymphatic system disorders	anaemia	97	1.47 (1.2 - 1.8)	4	0.74 (0.28 - 1.96)*	25	0.47 (0.32 - 0.7)*	2	0.93 (0.23 - 3.72)*	130	1.8 (1.51 - 2.14)
Metabolism and nutrition disorders	dehydration	80	1.39 (1.11 - 1.73)	10	2.12 (1.14 - 3.94)	32	0.7 (0.49 - 0.99)	/	/	117	1.85 (1.54 - 2.23)
Surgical and medical procedures	hospitalisation	67	1.4 (1.1 - 1.78)	6	1.52 (0.68 - 3.4)	21	0.55 (0.36 - 0.84)*	1	0.64 (0.09 - 4.55)*	136	2.63 (2.21 - 3.12)

Footnotes: SOC, system organ class; PT, preferred term; ROR, reporting odds ratio; CI, confidence interval; To obtain robust results and reduce the false positive signals, signal values were only calculated for complications with at least 3 records. A signal was defined as both χ2 > 4 and lower 95% CI > 1. Negative signals were highlighted in white with*.

### General disorders and administration site conditions

Injection site pain was the most frequently reported PT(ROR = 10.09, 95% CI [9.66–10.54], n = 3,134), with etanercept (ROR = 16.41, 95% CI [15.51–17.35], n = 1,569) and adalimumab (ROR = 12.46, 95% CI [11.78–13.17], n = 1,546) collectively accounting for 99.39% of cases (3,115/3,134). The second most reported PT was Pyrexia (ROR = 3.5, 95% CI [1.07–1.2], n = 1,358), where adalimumab (ROR = 1.36, 95% CI [1.24–1.48], n = 538) and etanercept (ROR = 1.49, 95% CI [1.35–1.63], n = 463) contributed to 73.71% of cases (1,001/1,358). Notably, infliximab showed no significant risk signal despite 326 reported cases.Within the same SOC, the strongest ROR signal was observed for Injection site warmth(ROR = 26.72, 95% CI [21.88–32.63], n = 233), with etanercept demonstrating the highest signal intensity among TNF-α inhibitors (ROR = 66.83, 95% CI [54.86–81.42], n = 187). Injection site pruritus (ROR = 19.81, 95% CI [17.61–22.28], n = 569) also displayed a pronounced signal, particularly for etanercept (ROR = 41.58, 95% CI [36.74–47.04], n = 394), which surpassed other TNF-α inhibitors in signal magnitude.

### Gastrointestinal disorders

Abdominal pain emerged as the most frequently reported PT(ROR = 2.85, 95% CI [2.69–3.03], n = 1,308), with infliximab (ROR = 4.56, 95% CI [4.24–4.91], n = 783) and adalimumab (ROR = 2.65, 95% CI [2.41–2.92], n = 430) accounting for 92.74% of cases (1,213/1,308). While 95 cases were reported for certolizumab, etanercept, and golimumab, no significant risk signals were detected. Subsequent high-frequency PTs included haematochezia (ROR = 3.5, 95% CI [13.84–16.19], n = 1,127) and diarrhoea (ROR = 1.53, 95% CI [1.43–1.63], n = 1,005).Within this SOC, infliximab and adalimumab generated the highest PT counts for gastrointestinal disorders. Adalimumab demonstrated strong risk signals across all five analyzed PTs. Compared to other TNF-α inhibitors, infliximab exhibited significantly stronger signals for frequent bowel movements (ROR = 41.4, 95% CI [35.7–48.01], n = 313), haematochezia (ROR = 26.37, 95% CI [24.27–28.66], n = 839)and abdominal pain (ROR = 4.56, 95% CI [4.24–4.91], n = 783).

### Infections and infestations

Nasopharyngitis (ROR = 3.15, 95% CI [2.89–3.42], n = 629) was the most frequently reported PT, with risk signals detected across all five TNF-α inhibitors. Etanercept demonstrated the highest case count and strongest signal intensity (ROR = 5.35, 95% CI [4.75–6.03], n = 292). The most potent signal within this SOC was observed for clostridium difficile infection (ROR = 8.44, 95% CI [7.3–9.75], n = 266), where infliximab exhibited significantly stronger signal strength compared to other TNF-α inhibitors (ROR = 16.72, 95% CI [14.29–19.56], n = 206), accounting for 77.4% of cases.

### Other SOC

All TNF-α inhibitors demonstrated strong signals for uveitis (ROR = 23.89, 95% CI [20.16–28.22], n = 308), particularly adalimumab (ROR = 20.96, 95% CI [17.82–24.65], n = 145) and golimumab (ROR = 52.08, 95% CI [31.07–87.33], n = 15). Off label use emerged as the most frequently reported PT(ROR = 2.07, 95% CI [2.01–2.14], n = 4,805), with infliximab (ROR = 4.56, 95% CI [3.02–3.28], n = 2,650) and adalimumab (ROR = 2.34, 95% CI [2.22–2.47], n = 1,459) accounting for 85.52% of cases (4,109/4,805).

### Age subgroup analysis

**Fig 5:**
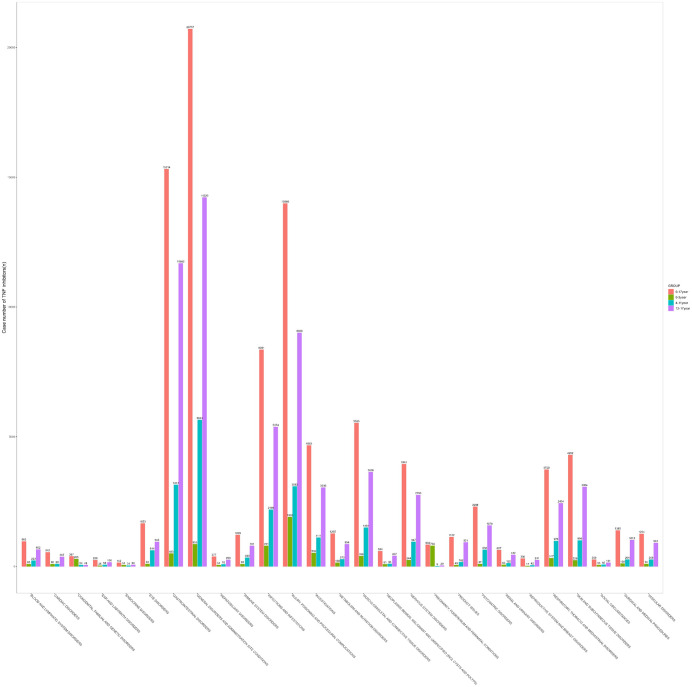
Proportion of Cases Involving Risk-Prioritized PTs at the SOC Level. Results represent the ratio of cases associated with risk signals to the total case count. Subgroup analyses stratified pediatric patients into three age cohorts: toddlers group (0–3 years), younger children group (4–11 years), and adolescents group (12–17 years).

Age-subgroup analysis revealed distinct AE patterns across developmental stages (Fig 5). The adolescents groups(12–17 years) exhibited the highest proportion of gastrointestinal disorders (17.97%), significantly exceeding rates in younger children groups (4–11 years: 13.66%) and toddlers groups (0–3 years: 6.17%). This age group also demonstrated the strongest risk signals for gastrointestinal events (ROR = 2.96, 95% CI [2.90–3.03], n = 11,680). Conversely, general disorders and administration site conditions were most prevalent in younger children groups (4–11 years: 24.55%), exceeding rates in toddlers groups (0–3 years: 10.68%) and adolescents groups (12–17 years: 21.88%). For injury, poisoning and procedural complications, toddlers groups (0–3 years) showed the highest proportion (23.82%), significantly outpacing other age groups.

### Time-to-onset analysis of adverse events

**Fig 6:**
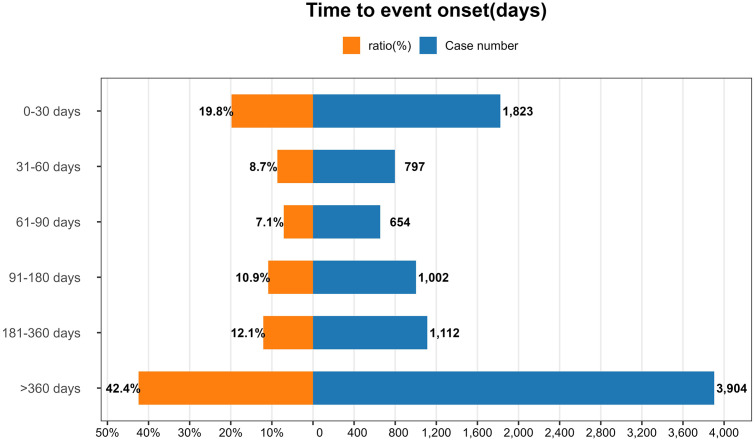
Time distribution of adverse events induced by TNF-α inhibitors in children. The right axis shows the number of reported cases for each time interval, while the left axis represents the percentage ratio of total cases.

Among pediatric reports containing target AEs, 9,202 cases (26.73% of target AE reports) were excluded due to missing event timelines. The median time-to-onset for TNF-α inhibitors-associated AEs across all agents was 235 days (IQR46–832 days), with adalimumab (Median 87 days, IQR 25–325 days), certolizumab (Median 130 days, IQR 35–466 days), etanercept (Median 22 days, IQR 22–393 days), golimumab (Median 204 days, IQR 68.75–522.75 days), and infliximab (Median 572 days, IQR 154.5–1,323 days). As demonstrated in Fig 6,the majority of AEs occurred within the first month of TNF-α inhibitors therapy, with a pronounced decline in months 2–3, emphasizing the imperative for intensive safety surveillance during treatment induction. Conversely, AE incidence resurged as a late-phase risk cluster beyond one year of treatment, highlighting the necessity for extended clinical follow-up in this patient population.

### Sensitivity analysis

Median RORs were 3.6 vs 2.9, respectively. A paired Wilcoxon signed-rank test showed a small but statistically significant difference (median difference 0.47; Z = 3.21, p = 0.001). The absolute discrepancy is negligible compared with the order-of-magnitude differences in RORs, and 95% CIs overlap extensively for the vast majority of PTs. More importantly, no systematic directional inflation in the early period was observed; several gastrointestinal and laboratory signals actually exhibited higher RORs in the later period. We therefore conclude that temporal exposure length has not materially biased our comparative signal rankings. See Table S2 for details.

## Discussion

TNF-α inhibitors represent cornerstone therapeutics for pediatric refractory autoimmune diseases, yet demand stringent adherence to approved indications, proactive surveillance of infection and malignancy risks, and personalized treatment regimens.Current guidelines recommend initiating biologic therapy after failure of conventional treatments such as methotrexate or glucocorticoids [9]. The use of TNF-α inhibitors in children serves as a critical therapeutic approach for refractory conditions, particularly in diseases such as JIA and pediatric IBD [10], which demonstrate higher rates of treatment resistance.

Among the TNF-α inhibitors analyzed, adalimumab [11], certolizumab [12], and etanercept [13] are approved by the FDA for polyarticular JIA in patients aged ≥2 years, while infliximab [14] is approved for pediatric Crohn’s disease in children ≥6 years. Golimumab [15] is currently approved exclusively for adult populations, with no pediatric approvals to date. Although multiple Phase III clinical trials have evaluated biologics in pediatric refractory autoimmune diseases—with detailed safety analyses during trial periods (Gerd Horneff et al., 2018 [16]; Daniel J. Lovell et al., 2008 [17]; Michael D. Kappelman et al., 2023 [18])—a critical gap persists in Phase IV post-marketing surveillance for long-term safety. Consequently, clinician awareness of both common and rare TNF-α inhibitors-associated AEs is essential for optimizing pediatric care quality.

In our analysis of 34,423 pediatric reports, we observed significant gender differences among the patients, with a notable predominance of females. This may reflect the higher incidence of autoimmune diseases, such as juvenile idiopathic arthritis and inflammatory bowel disease, in women [19]. Therefore, it is essential to enhance monitoring of female patients receiving TNF-α inhibitors in clinical practice. We also noted significant age-dependent differences across distinct age groups: infants (0–3 years), children (4–11 years), and adolescents (12–17 years). Each subgroup exhibited different patterns of AE reporting, highlighting the variations in drug response and adverse event occurrence across different age brackets. Specifically, adolescents (ages 12–17) accounted for 68.8% of reports, indicating their extensive use of these medications, which aligns with approved indications, such as adalimumab for moderate to severe hidradenitis suppurativa in patients aged 12 years and older, and for active Crohn’s disease in those above 6 years. In terms of severe adverse outcomes, comparisons between different TNF-α inhibitors revealed safety and tolerability differences, as we classified clinical outcomes related to death, life-threatening conditions, disability, and hospitalization as serious adverse events. Notably, adalimumab showed the highest proportion of serious outcomes (46.9%) among pediatric cases, warranting clinicians remain proactive in their surveillance strategies. As these age and gender dynamics emerge from our data, it is critical for healthcare providers to adjust their monitoring and treatment protocols accordingly, maintaining a focus on individualized treatment plans.

This study revealed a significantly lower risk signal detection rate (RSR = 18.85%) in pediatric populations compared to all-age populations (RSR = 21.03%), potentially attributable to lower medication exposure due to restricted pediatric indications [20]. Notably, etanercept exhibited an elevated RSR in children versus all-age populations, a phenomenon potentially linked to its unique receptor fusion protein structure [21], which may induce distinct immune responses.

At the SOC level, infectious events (19.84%) and injection site reactions (12.44%) emerged as the primary safety concerns in pediatric populations. Younger children groups (4–11years) exhibited a heightened susceptibility to infections compared to adolescent groups, a finding consistent with immature macrophage function and inefficient granuloma formation observed in pediatric populations, where TNF-α inhibition may further compromise antimicrobial defenses [4]. Frequent subcutaneous or intravenous administration was directly associated with an increased prevalence of localized reactions, exacerbated by the underdeveloped skin barrier function and a propensity for inflammatory dissemination in children [22]. Notably, infliximab exhibited disproportionately strong signals for clostridium difficile infection (ROR = 16.72, 95% CI 14.29–19.56) and hematochezia (ROR = 26.37, 95% CI 24.27–28.66), potentially attributable to its profound suppression of gut microbiota diversity, including the depletion of clostridiales [23]. Therefore, clinicians should consider stool testing and early empiric therapy in pediatric IBD patients presenting with diarrhea.

Age subgroup analysis identified distinct developmental risk patterns, indicating that adolescents (12–17 years) showed a 191% increase in the incidence of gastrointestinal disorders compared to toddlers (0–3 years) (17.97% vs. 6.17%), respectively. This increase aligns with murine data showing that estrogen withdrawal increases TNF-α–dependent intestinal permeability, whereas 17β-estradiol replacement restores barrier function, providing a mechanistic link between pubertal hormonal fluctuations and heightened susceptibility to anti-TNF–related gut injury [24,25].

This suggests a need for proactive gastrointestinal monitoring in adolescents patients receiving TNF-α inhibitors, including regular assessments for symptoms such as abdominal pain, diarrhea, or weight loss. Conversely, younger children (4–11 years) demonstrated higher rates of systemic reactions (24.55%), highlighting the necessity for age-specific pharmacovigilance protocols. For younger children, clinical strategies may involve closer monitoring for systemic reactions, particularly during the initiation of therapy. Such targeted approaches not only improve patient safety but also enhance overall treatment outcomes in pediatric populations. By recognizing these age-related differences, we emphasize the importance of individualized patient care and the need for healthcare providers to adapt their monitoring and treatment protocols accordingly.

High-intensity safety signals, particularly increased bowel frequency (ROR = 41.4) and injection site warmth (ROR = 66.83), necessitate prioritized integration into pediatric pharmacovigilance frameworks. The predominance of injection site reactions with etanercept (67.3% of cases) correlates strongly with its subcutaneous administration frequency, underscoring the need for alternate delivery strategies in chronic pediatric use. while adalimumab’s reformulated citrate-free formulation demonstrated 40% reduction in localized AEs [26], providing clear direction for pediatric-focused drug delivery optimization.

This study corroborates the infection risk warnings for TNF-α inhibitors previously reported by Krabbe [27], while uniquely identifying clostridioides difficile infection as a predominant pediatric safety concern, contrasting with prior adult-focused studies emphasizing respiratory infections. Although clostridioides difficile infection is well described in adults with IBD who receive infliximab [28], our padiatric disproportionality analysis now identifies it as a dominant safety signal (ROR = 16.72). This finding is consistent with a recent single-centre padiatric IBD cohort [29], and the 206 reports of *C. difficile* infection retrieved from FAERS highlight the need for heightened clinical vigilance in children initiating anti-TNF therapy.

At the SOC level, pediatric gastrointestinal reaction rates significantly exceeded all-age populations 20.53% (0.81% vs. 0.67%), aligning with earlier pediatric pharmacovigilance findings [30]. These results demonstrate that extrapolating adult safety data to pediatric populations may introduce significant bias, highlighting the necessity for age-specific pharmacovigilance frameworks.

Overall, our study showed significant heterogeneity in the safety profiles of TNF-α inhibitors among pediatric populations. Through comprehensive characterization of these drugs based on the FAERS, we identified novel, severe, or uncommon AE signals. While the spontaneous reporting of FAERS exhibits inherent biases (e.g., underreporting, confounding by indication), comparative disproportionality analysis across TNF-α inhibitors mitigates confounding through internal benchmarking, enabling relative risk differentiation.

Our findings highlight the importance of interpreting disproportionality signals, such as the ROR, alongside AE frequencies and clinical plausibility. For example, golimumab showed the strongest ROR signal for uveitis (ROR = 52.09, 95% CI: 31.07–87.33), yet its low report count (n = 15) suggests a rare but potentially specific risk. Conversely, adalimumab demonstrated moderate ROR despite a higher number of uveitis reports (n = 145), likely reflecting its widespread use rather than an elevated per-exposure risk. To address these complexities, we advocate for a multidimensional analytical framework that integrates: (1) absolute AE frequencies to distinguish rare yet significant signals from common ones; (2) external prescription data to account for variations in drug exposure [31]; (3) mechanistic evidence, such as golimumab’s monoclonal antibody structure, which may predispose to uveitis compared to etanercept’s fusion protein mechanism [21].

Furthermore, several limitations of spontaneous reporting systems should be acknowledged. First, spontaneous reporting systems are influenced by under-reporting, selective reporting, and stimulated reporting, which make signals look stronger or weaker than it really is. Second, ROR indicates statistical associations, not causal relationships, we still need epidemiologic or prospective studies to confirm causality. Third, FAERS contains mainly U.S. and some European reports; therefore, our findings may not generalize to populations with different prescribing patterns, ethnic backgrounds, or health systems. Finally, the absence of precise denominator data (i.e., exact prescription numbers) precludes the calculation of incidence rates. Thus, the results should be interpreted as reporting tendencies rather than absolute risk.

As the first large-scale pharmacovigilance study focusing on pediatric TNF-α inhibitor safety using real-world data, these findings can provide evidence for optimizing therapeutic decision-making by contrasting agent-specific risk patterns, such as optimizing therapeutic decision-making by contrasting agent-specific risk patterns, or prioritizing age-stratified monitoring for high-intensity signals, guiding pediatric-focused drug delivery optimization. These results highlight the necessity for dedicated pediatric pharmacovigilance frameworks to address developmental-stage-specific vulnerabilities, rather than extrapolating safety data from adult populations.

## Conclusion

This large-scale pharmacovigilance study systematically characterized the safety profile of TNF-α inhibitors in pediatric populations using real-world data from FAERS. Multiple injection site reactions, infectious events and gastrointestinal disorders as common ADR. Uveitis was detected across all TNF-α inhibitors, with signal intensity varying by agent. Age-stratified analyses revealed differential risk patterns, including significantly elevated gastrointestinal risks in adolescents and systemic reactions in younger children. Future research should prioritize observational studies and multicenter prospective cohorts to validate critical findings, while integrating therapeutic drug monitoring (TDM) to elucidate dose-response relationships—a limitation not addressed in the current analysis.

Specific next steps may include conducting pediatric trials that incorporate microbiome monitoring to better understand the impact of TNF-α inhibitors on gut microbiota and infection risks. Additionally, developing pediatric-specific safety checklists (e.g., routine *C. difficile* screening in infliximab-treated IBD patients) would be beneficial. Furthermore, public health reporting amendments should be considered to capture pediatric-specific events more effectively. These efforts will strengthen causal inference and inform age-specific risk mitigation strategies for TNF-α inhibitors therapy in children. These efforts will strengthen causal inference and inform age-specific risk mitigation strategies for TNF-α inhibitors therapy in children. Our findings support the need for pediatric-specific regulatory guidance for TNF-α inhibitor safety monitoring.

## Supporting information

S1 Table Complete list of 852 pediatric adverse event signals by SOC for five TNF-α inhibitors from the FAERS database (Q1 2004–Q3 2024). This table presents the AE signals identified in children treated with TNF-α inhibitors, including the event PT, the number of reported cases (n), the ROR indicating the strength of the signal, and the corresponding 95% CI.
(XLSX)

S2 Table Sensitivity analysis of the 50 most frequently reported PTs: comparison of RORs between early-phase (Q1 2004–Q4 2013) and recent-phase (Q1 2014–Q3 2024) post-marketing surveillance periods. Footnote: Δ = ROR(041–134) − ROR(141–243). “Overlap of 95% CI” indicates whether the two confidence intervals overlap (Yes/No).
(XLSX)
